# SIRT6 Suppresses NFATc4 Expression and Activation in Cardiomyocyte Hypertrophy

**DOI:** 10.3389/fphar.2018.01519

**Published:** 2019-01-08

**Authors:** Zhenzhen Li, Xiaoying Zhang, Zhen Guo, Yao Zhong, Panxia Wang, Jingyan Li, Zhuoming Li, Peiqing Liu

**Affiliations:** ^1^Department of Pharmacology and Toxicology, School of Pharmaceutical Sciences, Sun Yat-sen University, Guangzhou, China; ^2^National and Local United Engineering Lab of Druggability and New Drugs Evaluation, School of Pharmaceutical Sciences, Sun Yat-sen University, Guangzhou, China; ^3^Guangdong Provincial Key Laboratory of New Drug Design and Evaluation, School of Pharmaceutical Sciences, Sun Yat-sen University, Guangzhou, China; ^4^Department of Pharmacology, School of Medicine, Xizang Minzu University, Shaanxi, China; ^5^Department of Cardiology, Third People’s Hospital of Dongguan, Dongguan, China

**Keywords:** cardiomyocyte hypertrophy, SIRT6, deacetylase activity, NFATc4, BNP

## Abstract

NFATc4, a member from the Nuclear Factor of Activated T cells (NFATs) transcription factor family, plays a pivotal role in the development of cardiac hypertrophy. NFATc4 is dephosphorylated by calcineurin and translocated from the cytoplasm to the nucleus to regulate the expression of hypertrophic genes, like brain natriuretic polypeptide (BNP). The present study identified SIRT6, an important subtype of NAD^+^ dependent class III histone deacetylase, to be a negative regulator of NFATc4 in cardiomyocyte hypertrophy. In phenylephrine (PE)-induced hypertrophic cardiomyocyte model, overexpression of SIRT6 by adenovirus infection or by plasmid transfection repressed the protein and mRNA expressions of NFATc4, elevated its phosphorylation level, prevented its nuclear accumulation, subsequently suppressed its transcriptional activity and downregulated its target gene BNP. By contrast, mutant of SIRT6 without deacetylase activity (H133Y) did not demonstrate these effects, suggesting that the inhibitory effect of SIRT6 on NFATc4 was dependent on its deacetylase activity. Moreover, the effect of SIRT6 overexpression on repressing BNP expression was reversed by NFATc4 replenishment, whereas the effect of SIRT6 deficiency on upregulating BNP was recovered by NFATc4 silencing. Mechanistically, interactions between SIRT6 and NFATc4 might possibly facilitate the deacetylation of NFATc4 by SIRT6, thereby preventing the activation of NFATc4. In conclusion, the present study reveals that SIRT6 suppresses the expression and activation of NFATc4. These findings provide more evidences of the anti-hypertrophic effect of SIRT6 and suggest SIRT6 as a potential therapeutic target for cardiac hypertrophy.

## Introduction

Cardiac hypertrophy is an adaptive response of the heart to various physiological or pathological stimuli. It is initially a compensatory mechanism to maintain cardiac output. However, prolonged hypertrophy ultimately progresses to arrhythmia, heart failure, or sudden death (Frey et al., 2004; Rohini et al., 2010). Cardiac hypertrophy is characterized by an increase in cell size and protein synthesis, an enhancement of sarcomeric organization, and reactivation of fetal genes, including atrial natriuretic peptide (ANF) and brain natriuretic peptide (BNP), and β-myosin heavy chain (β-MHC) (Cotecchia et al., 2015). A number of complex prohypertrophy signaling cascades have been identified to be involved in the regulation of cardiac hypertrophic response, whereas potential antihypertrophy signaling pathways have been discovered as well, comprising a complex signaling network. Identifying the crosstalk between these pro-hypertrophic and anti-hypertrophic signals helps to understand the molecular and cellular mechanisms underlying the development of cardiac hypertrophy and heart failure (Govindsamy et al., 2018). Augmenting the inhibitory signaling pathways might suggest a promising strategy for treatment of cardiac hypertrophy.

NFATc4, a member of nuclear factor of activated T cells (NFAT) transcription factor family, has been well-documented for its involvement in several diseases including cardiac hypertrophy, by regulating various target genes (Horsley and Pavlath, 2002; Li et al., 2016a, 2017; Zhang and Storey, 2016; Wang et al., 2017; Sharma et al., 2018). Similar as other NFAT subtypes, NFATc4 is dephosphorylated and activated by CaN (van Middendorp et al., 2017). After dephosphorylation, NFATc4 is transported from the cytoplasm to the nucleus and binds to target DNA to regulate the expression of hypertrophic genes, in particular the hypertrophic marker BNP (Molkentin et al., 1998; Crabtree, 1999; Liu et al., 2015; Li et al., 2016a, 2017). In addition, NFATc4 interacts with other hypertrophic signaling pathways, such as GATA-4, MAPK, GSK3, p38, and JNK (Coleman et al., 2015). Thus, CaN-NFAT signaling pathway has been regarded as a critical pathway that induces cardiac hypertrophy (Molkentin et al., 1998; Zhao et al., 2016).

Sirtuins, first discovered in yeast as silent information regulator 2 (Sir2), catalyzes NAD^+^-dependent histone and non-histone deacetylation and thus participates in many pathophysiological processes (Heineke and Molkentin, 2006; Sosnowska et al., 2017; Tasselli et al., 2017). The family of sirtuins contains seven subunits in mammals (SIRT1-SIRT7) that share a conserved core catalytic domain but differ in their cellular localization and tissue distribution. Among all, the nuclear-distributed SIRT6 has recently attracted numerous attentions for its critical role in regulating gene stability, metabolism, stress response, and aging (Mendes et al., 2017). Previous studies in our laboratory and others have revealed that SIRT6 can prevent and inhibit cardiac hypertrophy (Sundaresan et al., 2012; Yu et al., 2013; Shen et al., 2016; Zhang et al., 2016). SIRT6 suppresses cardiomyocyte hypertrophy via interaction with nuclear factor kappa B (NF-κB) catalytic subunit p65 to inhibit its transcriptional activity (Yu et al., 2013), via interaction with c-Jun and deacetylation of histone 3 at Lys9 (H3K9) to repress insulin-like growth factor signaling (Sundaresan et al., 2012), via repression of STAT3 (signal transducer and activator of transcription 3) (Zhang et al., 2016), and via decreasing the acetylase p300 expression by promoting its degradation (Shen et al., 2016). All these effects of SIRT6 are dependent on its deacetylase activity. The decrease of SIRT6 deacetylase activity in response to pressure overload or hypertrophic stimuli such as angiotensin II, isoprenaline and PE contributes to the pathogenesis of cardiac hypertrophy, although its expression is not altered (Cai et al., 2012; Yu et al., 2013; Lu et al., 2016; Shen et al., 2016; Zhang et al., 2016).

In this study, we identify that SIRT6 could suppress CaN-NFAT signaling pathway in cardiomyocyte hypertrophy. This mechanism provides a novel insight into the anti-hypertrophic effect of SIRT6 and suggests SIRT6 as a potential therapeutic target for cardiac hypertrophy.

## Materials and Methods

### Antibodies and Reagents

Anti-NFATc4 (diluted 1:200, sc-13036), anti-phospho-NFATc4 (diluted 1:100, sc-32630) polyclonal antibodies were purchased from Santa Cruz Biotechnology Inc. (Santa Cruz, CA, United States). Anti-SIRT6 (diluted 1:1000, 12486) antibody was bought from Cell Signaling Technology (Beverly, MA, United States). Anti-CaN A (diluted 1:1000, ab90540) antibody was purchased from Abcam (Cambridge, MA, United States). Anti-p38 (diluted 1:1000, 14064-1-AP) antibody was bought from Proteintech (Rosemont, IL, United States). Anti-α-tubulin (diluted 1:1000, T9026) and anti-Lamin B1 (diluted 1:1000, SAB1306342) antibodies were obtained from Sigma (St. Louis, MO, United States). Lipofectamine 2000 was obtained from Invitrogen (Carlsbad, CA, United States). PE was obtained from Tocris Bioscience (Bristol, United Kingdom). Rhodamine phalloidin and 4′6-diamidino-2-phenylindole (DAPI) were purchased from Invitrogen (Carlsbad, CA, United States). Other reagents were from Sigma-Aldrich unless otherwise stated.

### Primary Culture of Neonatal Rat Cardiomyocytes

The primary culture of NRCMs were prepared as previously described (Yu et al., 2013). NRCMs were isolated from the ventricles of 1 to 3-day-old SD rats. Briefly, hearts were removed immediately and kept in cold phosphate-buffered saline (PBS), and the minced ventricles were dispersed at 37°C in 0.08% trypsin solution approximately 12–14 times for 5 min each time. Cell suspension from each digestion was gathered. Finally, the cells were harvested by centrifugation for 8 min at 1400 × *g* and then suspended in Dulbecco’s modified Eagle’s medium (DMEM, Gibco, BRL Co., Ltd., United States) supplemented with 10% fetal bovine serum (FBS). The suspensions were plated in culture flasks for 1h at 37°C in a humidified atmosphere (5% CO_2_ and 95% air). NRCMs were seeded onto culture dishes in DMEM supplemented with 10% FBS and 5-bromodeoxyuridine (0.1 mM). PE, a α1 adrenergic receptor agonist, is commonly used to induce cardiomyocyte hypertrophy (Appert-Collin et al., 2007). The NRCMs were treated with 100 mM PE for 6, 12, or 24 h to induce hypertrophy.

### Animal Model, Chocardiography, and Morphometric Measurements

Sprague-Dawley rats (male, 200–220 g, SPF grade, Certification No. 44005800006455) were obtained from the Experimental Animal Center of Sun Yat-sen University (Guangzhou, China). The animal experiments were approved by the Research Ethics Committee of Sun Yat-sen University and were in accordance with the Guide for the Care and Use of Laboratory Animals (NIH Publication No 85-23, revised 1996). AAC surgery was conducted as previously described (Yu et al., 2013). Briefly, rats were randomly divided into two groups (the Sham group and the AAC group) and anesthetized with 10% chloral hydrate (350 mg/kg, *ip*). The adequacy of anesthesia was monitored by evaluating and recording body temperature, cardiac and respiratory rates and patterns, muscle relaxation, and lash reflex. Under sterile conditions, the abdominal aorta above the kidneys was exposed through a midline abdominal incision and constricted at 4–5 cm above the suprarenal artery with a 5–0 silk suture that was tied around both the aorta and a blunted 22-gauge needle. The needle was promptly removed after constriction. The Sham group underwent a similar procedure without banding the aorta. After the operation, the surgical wound was sutured, and gentamicin was given to prevent infection. Eight weeks after AAC surgery, two-dimensional-guided M-mode echocardiography was performed by a Technos MPX ultrasound system (ESAOTE, SpAESAOTE SpA, Italy) (Zhou et al., 2006). Afterward, the rats were sacrificed and then their hearts were quickly removed for trimming the left ventricles. 5-μm-thick histological cross sections of the heart tissues were stained with HE for morphometric measurement.

### Western Blot Analysis

Western blotting was performed as previously described (Guo et al., 2015). Proteins of cultured cells or rat left ventricular tissues were harvested using RIPA lysis buffer (Beyotime Nantong, Jiangsu, China) containing a protease inhibitor cocktail (Sigma-Aldrich, St. Louis, MO, United States) on ice. Nuclear extracts were prepared using the CelLytic Nuclear Extraction Kit (Sigma-Aldrich, St. Louis, MO, United States), according to the manufacturer’s instructions. Protein concentrations were measured using a bicinchoninic acid (BCA) Protein Assay Kit (Pierce, Rockford, IL, United States). Equal amount of proteins (35 μg of total proteins or 15 μg of nuclear proteins) were separated by 8∼12% sodium dodecyl sulfate-polyacrylamide gel electrophoresis (SDS-PAGE) and then transferred to polyvinylidene fluoride (PVDF) membranes (EMD Millipore Corporation, Billerica, MA, United States). The membranes were incubated with primary antibodies over night at 4°C, followed by incubating with appropriate horseradish peroxidase-conjugated secondary antibodies at room temperature for 1 h. Blots were developed with enhanced chemiluminescence reagent (Pierce, Rockford, IL, United States) and detected by the LAS4000 imager (GE Healthcare, Waukesha, WI, United States). The intensities of the blots were quantified by the Quantity One (Bio-Rad) software. α-tubulin was used as an internal control for total proteins, and Lamin B1 was used for normalizing nuclear proteins.

### RNA Extraction and Quantitative Real-Time Polymerase Chain Reaction (qRT-PCR)

Total RNA from heart tissues or cultured cells was extracted using Trizol Reagent (Invitrogen, Carlsbad, CA, United States) following the manufacturer’s instruction. A total of 5 μg RNA were reversely transcribed to first strand cDNA using two-step RT kit (Thermo, Fisher Scientific, Rockford, IL, United States). The mRNA levels of the target genes were determined using the SYBR Green Quantitative PCR kit (TOYOBO, Japan) on the Real-time Thermo Cycler (Thermo Fisher Scientific, Rockford, IL, United States) as previously described (Guo et al., 2018). Rat-specific primer for miR-133a and miR-29a-3p were purchased from Ribobio Co., Ltd. (Guangzhou, China). Rat-specific primers for BNP, SIRT6, NFATc4, and β-actin (listed in Supplementary Table S1) were synthesized by Sangon Biotech Co., Ltd. (Shanghai, China). U6 and β-actin were used as an internal control.

### Measurement of Cell Surface Area

NRCMs cultured in 48-well plates were fixed with 4% (w/v) paraformaldehyde in phosphate-buffered saline (PBS) for 15 min at room temperature or overnight at 4°C, followed by 0.5% Triton-100 treatment for 10 min. Then the cell incubation with 0.1% (v/v) rhodamine-phalloidin for 30 min, were washed with PBS three times and counterstained with DAPI. Images of the NRCMs were detected by the High Content Screening system (ArrayScanVTI, Thermo Fisher Scientific, Rockford, IL, United States). The cell surface area from randomly selected fields (50 for each group) was determined using the built-in image analysis software.

### Adenovirus Vector

Recombinant adenovirus vectors expressing green fluorescent protein (Ad-GFP) and GFP-tagged SIRT6 (Ad-SIRT6), respectively, were purchased from Hanbio Technology (Shanghai, China). The viruses were expanded in HEK293A cells and purified with a virus purification kit (Biomiga, United States). The purified viruses were dialyzed in dilution buffer and stored at -80°C.

### Plasmid Transfection

Rat-SIRT6 (NM_001031649.1) gene was cloned into the pcDNA3.1+ vector by clone site *HindIII/XhoI* with flag tag and confirmed by DNA sequencing in Sangon Biotech Co., Ltd. (Shanghai, China). SIRT6 mutant (H133Y) was constructed using the *Fast* Mutagenesis System (TRANS, Beijing, China) according to the manufacturer’s instructions. The following primers were used for mutagenesis: forward, 5′-GGA CAA GCT GGC CGA GCT GTA CGG AAA CAT-5′; reverse, 5′-ACA GCT CGG CCA GCT TGT CCC TGG GGA A-3′. NRCMs were transiently transfected with 4 μg WT-SIRT6 and H133Y using 5 μL Lipofectamine 2000 (Invitrogen Carlsbad, CA, United States) according to the manufacturer’s instructions.

### RNA Interference

Small inference RNA (siRNA) targeting SIRT6, NFATc4, and NC siRNA were obtained from Genepharma (Shanghai, China). The sequences of the siRNAs are shown in Supplementary Table S2. NRCMs seeded in 35 mm dishes were transfected with 100 pmol of targeted siRNA or NC siRNA using 5 μL of Lipofectamine 2000 (Invitrogen, Carlsbad, CA, United States) according to the manufacturer’s instructions. NRCMs were transfected with SIRT6 and NFATc4 siRNAs for 48 h. The control groups were transfected with NC sequences.

### Immunofluorescence (IF) Assay

NRCMs seeded on coverslips were fixed with 4% paraformaldehyde for 10 min. After washing with PBS for three times, the cells were permeabilized with 0.3% TritonX-100 for 5 min and followed by incubation with blocking solution at room temperature for 1 h. The cells were further treated for 1 h with primary antibody against NFATc4 (diluted 1:50), and incubated with Alexa Fluor-labeled secondary antibody (diluted 1:200, Santa Cruz Biotechnology, United States). The coverslips were mounted with DAPI and were detected by a confocal microscope (LSM 710, Carl Zeiss, Jena, Germany).

### Luciferase Reporter Gene Assay

NFATc4 reporter gene plasmid (inserted sequence of AGCAAC) was constructed with pGL3-Basic and confirmed by DNA sequencing in Sangon Biotech Co., Ltd. (Shanghai, China). The promega Renilla Luciferase vector contained herpes simplex virus thymidine kinase promoter (pRL-TK) and NFATc4 reporter plasmids pGL3. Dual-luciferase reporter assay system were obtained from Promega (Madison, WI, United States). Cells were seeded at 1 × 10^5^ cells⋅well^-1^ into 48-well plates, and co-transfected with NFATc4 reporter plasmid (400 ng per well) and pRL-TK (80 ng per well) as internal control. Total amounts of transfected DNA were equalized by the addition of empty vector. After 6 h incubation, the cells were infected with Ad-SIRT6 or Ad-GFP, followed by PE stimulation. Luciferase activity was measured by dual-luciferase reporter assay system (Promega) and normalized by Renilla luciferase activity of pRL-TK.

### Immunoprecipitation

Experiments were performed as previously described. 200 μg total protein were incubated with anti-NFATc4 antibody (diluted 1:200) or rat normal IgG (used as a control) overnight at 4°C and then incubated with protein G/A agarose beads (Thermo Fisher Scientific, Rockford, IL, United States) for 4 h. Afterward, the beads were washed with lysis buffer for three times and then boiled in loading buffer for 5 min. The immunoprecipitated proteins were detected by Western blotting (Yuan et al., 2014).

### Statistical Analysis

The data are presented as the means ± SEM. Differences between two groups were analyzed with Student’s *t*-test. Statistical analysis among the various groups was conducted by one-way analysis of variance (ANOVA) with Tukey’s *post hoc* test. In all cases, a *P*-value <0.05 was considered statistically significant.

## Results

### Calcineurin/NFATc4 Signaling Pathway Was Activated in Cardiac Hypertrophy

**FIGURE 1:**
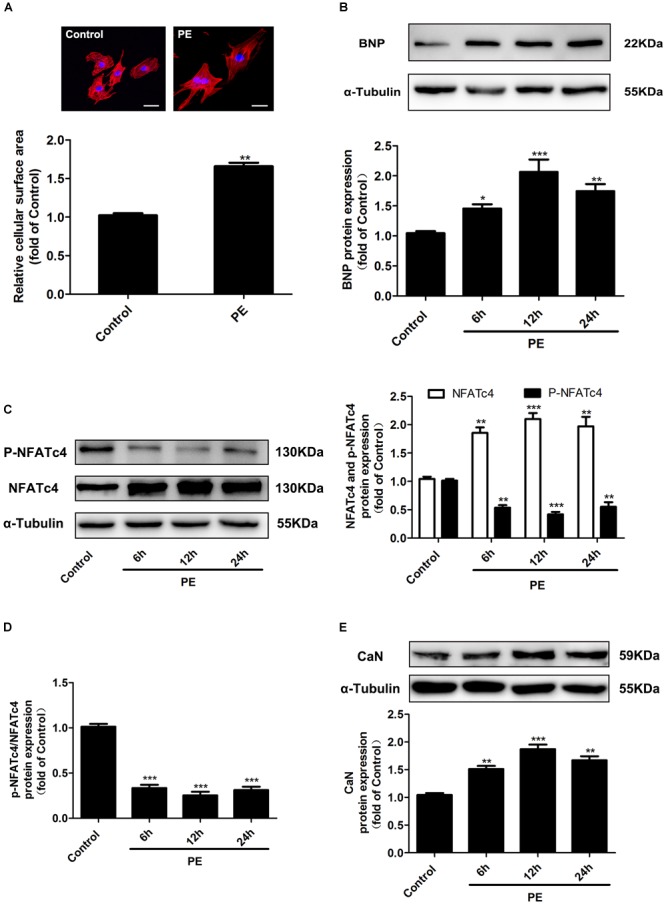
Changes of NFATc4 expression in PE-induced cardiac hypertrophy *in vitro*. Cultured NRCMs were incubated with 100 μM PE for the indicated durations. **(A)** The cell surface area was measured by rhodamine-phalloidin staining. Scale bar: 20 μm. Western blotting analysis was conducted to determine the protein expression of **(B)** BNP, **(C)** total NFATc4 and phosphorylated NFATc4, **(D)** p-NFATc4/NFATc4 ratio, and **(E)** calcineurin in NRCMs treated with 100 μM PE. The data were presented as mean ± SEM. ^∗^*P* < 0.05, ^∗∗^*P* < 0.01, ^∗∗∗^*P* < 0.001 vs. Control, *n* = 4.

The α-adrenoceptor agonist PE has been widely used to stimulate cardiomyocyte hypertrophy. In our study, treatment with 100 μM PE for 12 h significantly increased the cell surface area in NRCMs (Figure 1A). The expression of BNP, a marker of cardiac hypertrophy, was enhanced by 100 μM PE treated for 6, 12, and 24 h (Figure 1B). These results suggested that the PE-induced hypertrophic cardiomyocyte model was successfully established. In these hypertrophic cardiomyocytes, the protein expression of NFATc4 was significantly increased, whereas its phosphorylation level at Ser 168 and Ser 170 (p-NFATc4) was dramatically decreased (Figure 1C), resulting in a reduction of the p-NFATc4/NFATc4 ratio (Figure 1D). In addition, the protein expression of CaN was augmented by PE treatment (Figure 1E). Since NFATc4 is transported to the nucleus and activated following dephosphorylation by CaN, these observations thus indicate that the CaN/NFATc4 signaling pathway is activated in PE-induced cardiomyocyte hypertrophy.

**FIGURE 2:**
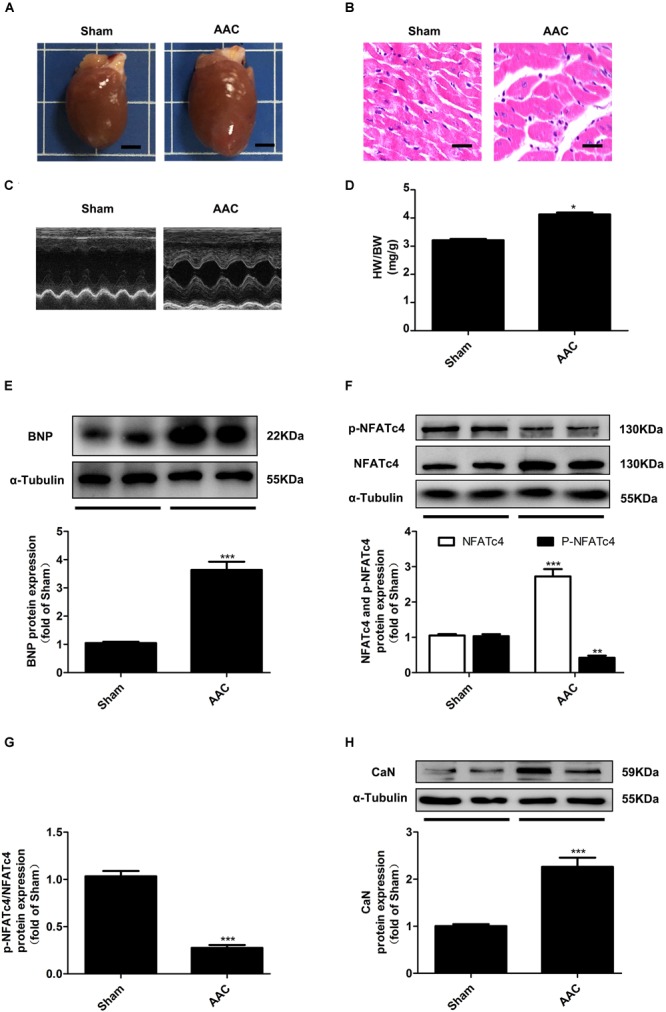
Changes of NFATc4 expression in pressure overload-induced cardiac hypertrophy *in vivo*. **(A)** Gross hearts from both AAC and Sham rats. Scale bar: 0.4 cm. **(B)** Histological analysis detected by hematoxylin and eosin staining. Scale bar: 10 μm. **(C)** Representative echocardiography analysis for both AAC and Sham rats. **(D)** The ratio of heart weight to body weight (HW/BW) were measured. Western blotting analysis was conducted to determine the protein expression of **(E)** BNP, **(F)** total NFATc4, phosphorylated NFATc4, **(G)** p-NFATc4/NFATc4 ratio, and **(H)** the protein expression of calcineurin in AAC and Sham rats. The data were presented as mean ± SEM. ^∗^*P* < 0.05, ^∗∗^*P* < 0.01, ^∗∗∗^*P* < 0.001 vs. Sham, *n* = 6.

To further confirm the changes of CaN/NFATc4 signaling pathway in cardiac hypertrophy *in vivo*, the rat cardiac hypertrophy model was induced by AAC operation. As shown in Figure 2A, the hearts from the AAC rats were larger than those from the Sham group. Pressure overload induced an increase in the myocyte diameter showed by HE staining (Figure 2B), an enhancement in LVAW and LVPW showed by echocardiography (Figure 2C and Supplementary Table S3), as well as an elevation in both the ratio of HW/BW and the protein expression of BNP (Figures 2D,E). In the hearts of AAC rats, the expressions of NFATc4 and CaN were upregulated, but the phosphorylation level of NFATc4 was attenuated (Figures 2F,H). As a result, the p-NFATc4/NFATc4 ratio was decreased (Figure 2G). These results are consistent with the *in vitro* observations showing that CaN/NFATc4 is activated during cardiac hypertrophy.

### SIRT6 Protected Against Cardiomyocyte Hypertrophy and Suppressed the Expression and Dephosphorylation of NFATc4 Dependent on Its Deacetylase Activity

**FIGURE 3:**
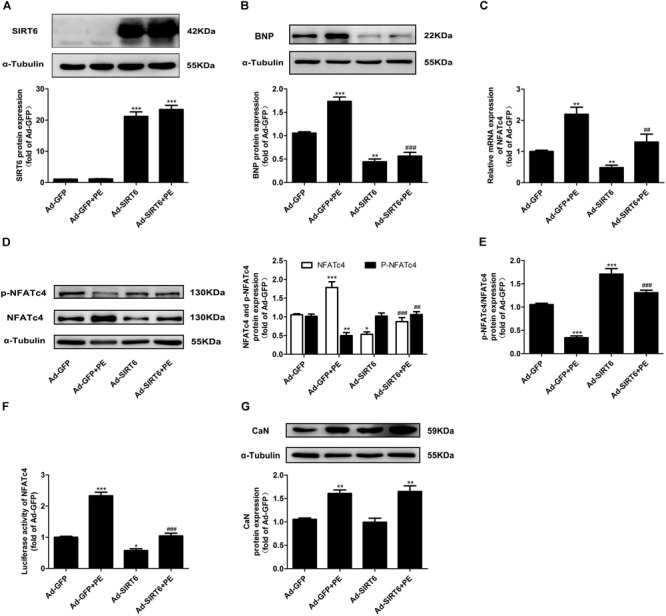
SIRT6 overexpression suppressed the expression level and dephosphorylation of NFATc4. NRCMs were infected with adenovirus vector encoding SIRT6 cDNA for 48 h. **(A,B)** The protein expression of SIRT6 and BNP were determined by Western blot analysis. **(C)** The mRNA expression of NFATc4 was determined by qRT-PCR. **(D)** Total NFATc4 and phosphorylated NFATc4 were investigated by Western blotting. **(E)** The ratio of p-NFATc4/NFATc4 was determined. **(F)** Dual luciferase reporter assays evaluating NFATc4-dependent transcriptional activity. **(G)** The protein expression of calcineurin was studied in NRCMs treated with 100 μM PE for 12 h. The data were presented as mean ± SEM. ^∗^*P* < 0.05, ^∗∗^*P* < 0.01, ^∗∗∗^*P* < 0.001 vs. Ad-GFP. ^##^*P* < 0.01, ^###^*P* < 0.001 vs. Ad-GFP + PE, *n* = 4.

To explore the regulatory role of SIRT6 in cardiac hypertrophy and CaN/NFATc4 pathway, NRCMs were infected with adenovirus vector encoding SIRT6 cDNA (Figure 3A). Ad-SIRT6 infection significantly reduced the protein expression of BNP, suggesting that SIRT6 protects the cardiomyocytes against PE-induced hypertrophy (Figure 3B). Ad-SIRT6 not only attenuated the mRNA and protein expression of NFATc4, but also reversed the decreased level of NFATc4 phosphorylation and the reduction of p-NFATc4/NFATc4 ratio induced by PE treatment (Figures 3C–E). Moreover, the effect of SIRT6 on the transcriptional activity of NFATc4 was evaluated by a dual luciferase reporter gene assay. Ad-SIRT6 repressed the transcriptional activity of NFATc4 in the presence or absence of PE (Figure 3F). However, SIRT6 overexpression did not alter the protein expression of CaN (Figure 3G).

**FIGURE 4:**
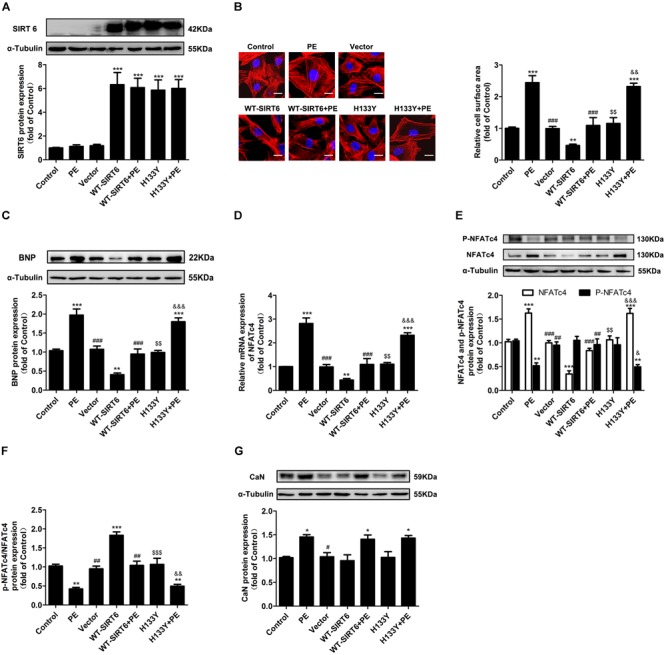
The effects of SIRT6 were dependent on deacetylase activity. NRCMs were transiently transfected with plasmid encoding the WT-SIRT6 and mutant of SIRT6 (H133Y) for 48 h followed by incubation with PE (100 μM for 12 h). **(A)** Western blot analysis was conducted to determine the protein expression of SIRT6. **(B)** The cell surface area was measured by rhodamine-phalloidin staining. Scale bar: 20 μm. **(C)** Western blot analysis was conducted to determine the protein expressions of BNP. **(D)** The mRNA expression of NFATc4 was determined by qRT-PCR. **(E)** The protein expression of total NFATc4, phosphorylated NFATc4 was determined by Western blotting. **(F)** The ratio of p-NFATc4/NFATc4 was calculated. **(G)** The protein expression of calcineurin was studied in NRCMs treated with 100 μM PE for 12 h. The data were presented as mean ± SEM. ^∗^*P* < 0.05, ^∗∗^*P* < 0.01, ^∗∗∗^*P* < 0.001 vs. Control. ^#^*P* < 0.05, ^##^*P* < 0.01, ^###^*P* < 0.001, vs. PE. ^$$^*P* < 0.01, ^$$$^*P* < 0.001, vs. WT-SIRT6. ^&^*P* < 0.05, ^&&^*P* < 0.01, ^&&&^*P* < 0.001, vs. WT-SIRT6 + PE. *n* = 4.

To further elucidate whether or not the effect of SIRT6 on NFATc4 is dependent on its deacetylase activity, we constructed the plasmids encoding the wildtype SIRT6 (WT-SIRT6) and mutant of SIRT6 without deacetylase activity (H133Y). First, to validate our method, we transfected NRCMs with GFP-SIRT1 plasmid by using Lipofectaminise 2000. The cells were observed under fluorescence microscope, which showed a high transfection efficiency (Supplementary Figure S3A). Consistent with the observations by Ad-SIRT6 infection, transfection of WT-SIRT6 decreased the cell surface area and BNP expression in NRCMs treated with or without PE (Figures 4A–C), downregulated the mRNA and protein expression of NFATc4 (Figures 4D,E), and recovered the repression of p-NFATc4 level and p-NFATc4/NFATc4 ratio caused by PE treatment (Figures 4E,F), without affecting CaN expression (Figure 4G). Unlike WT-SIRT6, transfection with the SIRT6 mutant H133Y did not ameliorate cardiomyocyte hypertrophy (Figures 4A–C), did not alter NFATc4 expression, the p-NFATc4 level or the pNFATc4/NFATc4 ratio (Figures 4D–F). These results indicate that the inhibitory effect of SIRT6 on NFATc4 expression and dephosphorylation is dependent on its deacetylase activity.

### SIRT6 Prevented PE-Induced Nuclear Translocation of NFATc4 Dependent on Its Deacetylase Activity

**FIGURE 5:**
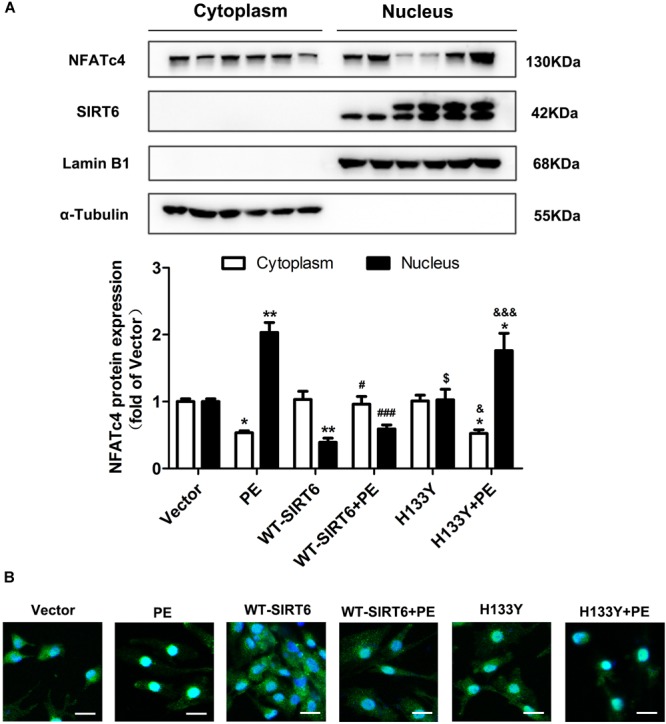
SIRT6 prevented PE-induced nuclear translocation of NFATc4 by interfering with calcineurin-NFATc4 interaction. NRCMs were transiently transfected with plasmid encoding the WT-SIRT6 and mutant of SIRT6 (H133Y) for 48 h followed by incubation with PE (100 μM for 12 h). **(A)** The protein expression of NFATc4 in the nucleus and cytoplasm was measured by Western blotting. **(B)** The presence of shuttling between the nucleus and cytoplasm of NFATc4 was observed by IF microscopy. The results were normalized to those of α-tubulin/LaminB1. The data were presented as mean ± SEM. ^∗^*P* < 0.05, ^∗∗^*P* < 0.01 vs. Vector. ^#^*P* < 0.05, ^###^*P* < 0.001 vs. PE. ^$^*P* < 0.05 vs. WT-SIRT6. ^&^*P* < 0.05, ^&&&^*P* < 0.001 vs. WT-SIRT6 + PE. *n* = 4.

The activation of NFATc4 requires dephosphorylation at Ser 168 and Ser 170 and subsequent translocation from cytoplasm to nucleus. Since the above results indicate that SIRT6 inhibits NFATc4 dephosphorylation, we further investigated whether or not the nuclear accumulation of NFATc4 is affected by SIRT6 overexpression. As shown by the Western blotting data, PE treatment augmented the expression of NFATc4 in the nuclear fractions of cardiomyocytes, but diminished that in the cytoplasmic fractions (Figure 5A). Overexpression of WT-SIRT6 significantly repressed the nuclear expression of NFATc4 in the presence or absence of PE, without affecting the cytoplasmic expression (Figure 5A). However, H133Y itself did not alter NFATc4 expression in either the nuclear fraction or the cytoplasmic fraction. Additionally, H133Y did not influence the effect of PE (Figure 5A). Results of the immunofluorescence experiments further confirmed that WT-SIRT6 but not H133Y prevented PE-facilitated NFATc4 occupancy in nucleus (Figure 5B).

### SIRT6 Suppressed NFATc4 Dephosphorylation and Nuclear Accumulation via Deacetylating It

The dephosphorylation and translocation of NFATc4 relies on the activation of CaN. However, the above results indicated that WT-SIRT6 could not change the expression of CaN. This finding raises the hypothesis that SIRT6 might alter the kinase which enhances the phosphorylation of NFATc4 to prevent NFATc4 activation. To explore this hypothesis, the expression and phosphorylation of p38 were investigated. As shown in Supplementary Figure S1, overexpression of SIRT6 did not affect either the phosphorylation or the total expression of p38, thus eliminating the possibility that SIRT6 enhances NFATc4 dephosphorylation via activating its kinase p38.

**FIGURE 6:**
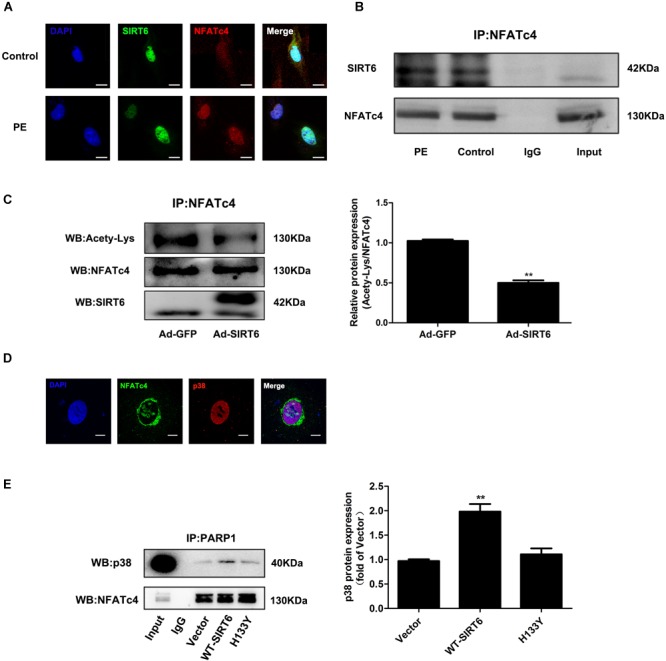
SIRT6 interacted with NFATc4 and deacetylated NFATc4 in NRCMs. **(A)** The intracellular colocalization of SIRT6 (Green) and NFATc4 (Red) in NRCMs treated with 100 μM PE for 12 h. **(B)** Co-immunoprecipitation shows SIRT6 interact with NFATc4. **(C)** The acetylated level of NFATc4 was detected in NRCMs infected with or without Ad-SIRT6. **(D)** The intracellular colocalization of NFATc4 (Green) and p38 (Red) in NRCMs. **(E)** Co-immunoprecipitation shows p38 interact with NFATc4 after SIRT6 and H133Y overexpression. The data were presented as mean ± SEM. ^∗∗^*P* < 0.01 vs. Ad-GFP. *n* = 4.

Interestingly, the results from immunofluorescence and Co-IP experiments demonstrated that there was interaction and co-localization between SIRT6 and NFATc4 (Figures 6A,B). In addition, SIRT6 overexpression reduced the acetylation level of NFATc4 (Figure 6C), suggesting that SIRT6 probably deacetylates NFATc4. These observations raise the hypothesis that NFATc4 might interact with SIRT6 and undergo deacetylation by SIRT6 in the nucleus, leading to a structural change of NFATc4, and facilitating its interaction with p38, finally enhancing its phosphorylation by p38 and nuclear export. Because of technical limitation, we failed to directly measure the changes of crystal structure of NFATc4. Instead, we examined the changes of interaction between NFATc4 and p38 after SIRT6 overexpression. The results showed that NFATc4 co-localized and interacted with p38 (Figures 6D,E), and that this interaction was enhanced by overexpression of SIRT6, but not H133Y (Figure 6E), supporting the above hypothesis.

### Suppression of NFATc4 Contributed to the Anti-hypertrophic Effect of SIRT6

**FIGURE 7:**
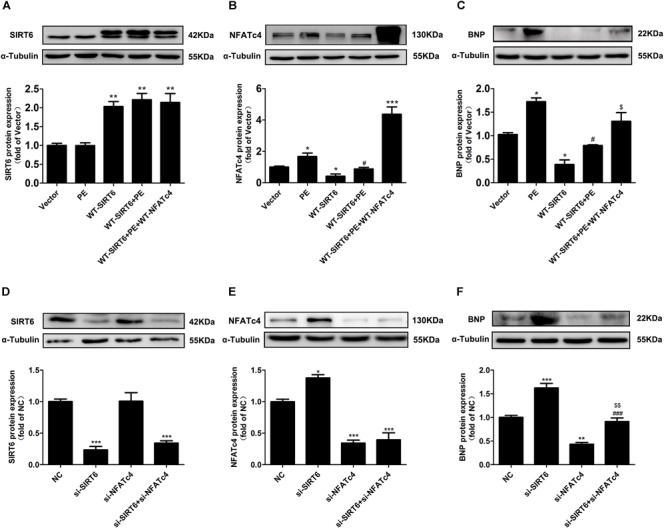
NFATc4 participated in the anti-hypertrophic effect of SIRT6. **(A–C)** Western blot analysis of the protein expression of SIRT6, NFATc4, and BNP in NRCMs transfected with or without WT-NFATc4, WT-SIRT6, and treated with 100 μM PE for 12 h. **(D–F)** The protein expression of SIRT6, NFATc4, and BNP in NRCMs transfected with siRNAs of SIRT6, NFATc4, or negative control (NC) for 48 h. The data were presented as mean ± SEM. ^∗^*P* < 0.05, ^∗∗^*P* < 0.01, ^∗∗∗^*P* < 0.001 vs. Vector or NC. ^#^*P* < 0.05, ^###^*P* < 0.001 vs. PE or si-SIRT6. ^$^*P* < 0.05, ^$$^*P* < 0.01, vs. WT-SIRT6 + PE or si-NFATc4. *n* = 4.

To elucidate that suppression of NFATc4 dephosphorylation and nuclear occupancy is involved in the anti-hypertrophic effect of SIRT6, NRCMs were transfected with WT-NFATc4 and WT-SIRT6, or treated with PE, alone or in combination (Figures 7A,B and Supplementary Figure S3B). Overexpression of NFATc4 significantly reversed the effect of SIRT6 on suppressing PE-induced upregulation of BNP (Figure 7C). In addition, endogenous NFATc4 and SIRT6 were knocked down by RNA interference in NRCMs (Figures 7D,E). Knockdown of NFATc4 recovered SIRT6 silencing-facilitated expression of BNP (Figure 7F). These observations suggest that suppression of NFATc4 contributes to the cardioprotective effect of SIRT6.

## Discussion

It is well established that activation of Ca^2+^-CaN-NFATc4 signaling pathway is associated with mechanical or agonist-induced cardiac hypertrophy (Molkentin et al., 1998; Bernt et al., 2016; Zhao et al., 2016; Lu et al., 2017; Parra and Rothermel, 2017; van Middendorp et al., 2017). In response to myocyte stretch or increased workloads of the heart, or stimuli by a variety of humoral factors including PE, angiotensin II, and endothelin-1, the elevation in intracellular Ca^2+^ initiates the activation of CaN, a ubiquitously expressed serine/threonine phosphatase, leading to dephosphorylation, and nuclear translocation of NFATc4, thereby facilitating its binding to DNA and interaction with other transcription factors, subsequently enhancing the expression of targeted hypertrophic genes (Molkentin et al., 1998; Coleman et al., 2015; Bernt et al., 2016; Zhao et al., 2016; Lu et al., 2017; Parra and Rothermel, 2017). In line with these findings, the present study demonstrated that expressions of NFATc4 and CaN were increased, whereas the phosphorylation level of NFATc4 was decreased in PE-induced *in vitro* cardiomyocyte hypertrophy model and AAC-induced *in vivo* cardiac hypertrophy model (Figures 1, 2).

The major novel finding of the present study is that SIRT6 acts as a negative regulator of CaN-NFATc4 signaling pathway in cardiomyocyte hypertrophy. As a promising therapeutic target for cardiovascular diseases (Vitiello et al., 2017), SIRT6 demonstrates potent anti-hypertrophic effect both *in vivo* and *in vitro* (Cai et al., 2012; Sundaresan et al., 2012; Yu et al., 2013; Lu et al., 2016; Shen et al., 2016; Zhang et al., 2016). The decrease in deacetylase activity of SIRT6 might contribute to the development of cardiac hypertrophy (Cai et al., 2012; Sundaresan et al., 2012; Yu et al., 2013; Lu et al., 2016; Shen et al., 2016; Zhang et al., 2016). Our study reveals that this anti-hypertrophic effect of SIRT6 involves suppression of CaN-NFATc4 signaling pathway. This conclusion is supported by the following observations: (1) Infection of Ad-SIRT6 or transfection of WT-SIRT6 plasmid reversed PE-induced upregulation and dephosphorylation of NFATc4, coordinating with the changes of cardiomyocyte surface area and hypertrophic gene BNP expression (Figures 3, 4); (2) SIRT6 overexpression inhibited PE-induced NFATc4 nuclear shuttling (Figure 5); (3) The effect of SIRT6 on repressing BNP expression was reversed by NFATc4 replenishment (Figures 7A–C); (4) SIRT6 deficiency-mediated BNP upregulation was recovered by NFATc4 silencing (Figures 7D–F). It is important to note that the effect of SIRT6 on inhibiting NFATc4 is dependent on its deacetylase activity, taken into account that the SIRT6 mutant H133Y lost the capability to suppress NFATc4 expression, dephosphorylation, and nuclear transport (Figures 4, 5). However, the present study indicates that SIRT6 did not alter protein expression level of CaN, although that was shown an elevation in PE-treated cardiomyocytes and in hearts of AAC rats (Figures 1E, 2H, 3G, 4G).

According to our observations, SIRT6 regulates NFATc4 by at least two ways. First, SIRT6 restrains NFATc4 expression at the transcriptional/post-transcriptional level, considering that overexpression of SIRT6 significantly decreased the mRNA and protein expression level of NFATc4 in the presence or absence of PE (Figures 3C,D, 4D,E). The mechanism by which SIRT6 suppresses NFATc4 expression remains unclear. As a class III histone lysine deacetylase, SIRT6 deacetylates the histones H3K9 and H3K56 to modify chromatin and regulate the transcription of a variety of genes (Wang et al., 2016; Agerholm et al., 2018; Wood et al., 2018). Accumulating evidences show that SIRT6 can transcriptionally regulate the expressions of Txnip (Qin et al., 2018), PDK4 (Khan et al., 2018), NF-κB target genes (Yu et al., 2013), and insulin-like growth factor signaling-related genes (Sundaresan et al., 2012) by catalyzing the deacetylation of H3K9 and/or H3K56 at their respective promoter regions. Therefore, it is possible that SIRT6 deacetylates potential histones at NFATc4 promoter region and thereby represses the transcription of NFATc4. This hypothesis is supported by the present results that the deacetylation activity of SIRT6 was required to attenuate NFATc4 expression (Figures 4D,E). In addition, the expression of NFATc4 is also repressed by microRNAs such as miR-133a and miR-29a-3p (Li et al., 2010, 2016b). Taken into considerations that SIRT6 plays an important role in regulating microRNAs (Fan et al., 2016; Kugel et al., 2016), it is hypothesized that SIRT6 suppresses NFATc4 expression via regulating miR-133a and miR-29a-3p. In order to validate this hypothesis, we have examined the expression of miR-133a and miR-29a-3p in SIRT6-overexpressed cells. The results showed that SIRT6 overexpression significantly increased the expression of miR-133a, but did not affect miR-29a-3p (Supplementary Figure S2). Thus, it is most likely that SIRT6 suppresses NFATc4 expression via augmenting the expression of miR-133a.

Second, SIRT6 inhibits NFATc4 activation by augmenting its phosphorylation level and preventing its nuclear accumulation. This conclusion is supported by the observations that the phosphorylated level of NFATc4 was increased (Figures 3D,E, 4E,F), but the nuclear expression was reduced after SIRT6 overexpression (Figure 5). The phosphorylation of NFATc4 is a balanced action between the phosphatase CaN (also known as type-2B phosphatase, PP2B) and the potential kinases. Since our results demonstrated that SIRT6 did not influence the expression of CaN (Figures 3G, 4G), it seems most likely that SIRT6 might have an impact on the kinases participating in NFATc4 phosphorylation. The mitogen-activated protein kinases (MAPK) are considered to be crucial co-regulators of NFAT family (Coleman et al., 2015). The MAPK signaling pathway is generally subclassified into three main branches consisting of p38, c-Jun N-terminal kinases (JNKs), and extracellular signal-regulated kinases (ERKs) (Kasuya et al., 2018). Among these three sub-families, JNK and p38 are capable of phosphorylating NFATs directly in their N-terminal regulatory domains, resulting in net inhibition of nuclear occupancy. JNK is responsible for phosphorylating NFATc1, NFATc2, and NFATc3, but not NFATc4 (Chow et al., 1997; Porter et al., 2000; Yang et al., 2002); by contrast, p38 is shown to directly phosphorylate NFATc1, NFATc2, and NFATc4, rather than NFATc3. Inhibition of p38 activates CaN-NFAT signaling to promote hypertrophic cardiomyopathy (Gomez del Arco et al., 2000; Porter et al., 2000; Yang et al., 2002). These studies elicit the hypothesis that SIRT6 regulates NFATc4 phosphorylation by activating p38. However, our results indicated that SIRT6 overexpression did not alter p38 expression or phosphorylation (Supplementary Figure S1), thus excluding the possibility that SIRT6 regulates NFATc4 phosphorylation and nuclear accumulation via p38 activation. Surprisingly, NFATfc4 was shown to co-localize and interact with SIRT6, especially when it was activated and transported to the nucleus by PE treatment (Figure 6). Therefore, it is possible that NFATc4 might interact with SIRT6 and undergo deacetylation by SIRT6 in the nucleus, leading to a structural change of NFATc4 and facilitating its interaction with p38, finally enhancing its phosphorylation and nuclear export. This possibility was supported by the observations that the interaction between NFATc4 and p38 was increased by overexpression of SIRT6 rather than H133Y, in the meantime when NFATc4 acetylation was abrogated (Figures 6C,E).

## Conclusion

In conclusion, the present study identifies SIRT6 as a negative regulator of NFATc4 in cardiomyocyte hypertrophy. SIRT6 can repress NFATc4 expression and prevent its dephosphorylation and nuclear accumulation, dependent on its deacetylase activity. These findings provide more evidences of the anti-hypertrophic effect of SIRT6, and further suggest that targeting activation of SIRT6 might be promising strategies for treating cardiac hypertrophy and heart failure.

## Author Contributions

ZL, XZ, ZL, and PL designed and conducted the study, analyzed the data, and prepared the manuscript. ZG and JL performed the animal experiments. PW and YZ collected and interpreted the data.

## Conflict of Interest Statement

The authors declare that the research was conducted in the absence of any commercial or financial relationships that could be construed as a potential conflict of interest.

## Abbreviations

AAC: abdominal aortic constriction
BNP: brain natriuretic polypeptide
BW: body weight
CaN: calcineurin
EF: ejection fraction
FS: fractional shortening
HE: hematoxylin-eosin
HW: heart weight
LVAW: left ventricular anterior wall thickness
LVID: left ventricular internal diameter
LVPW: left ventricular posterior wall thickness
NC: negative control
NFATc4: nuclear factor of activated T cells c4
NRCMs: neonatal rat cardiomyocytes
NS: normal saline
PBS: phosphate buffer solution
PE: phenylephrine
SD: Sprague-Dawley
siRNA: small-interfering RNA
SIRT6: Sirtuin 6
