# Supplementary material for: SIRT6 Suppresses NFATc4 Expression and Activation in Cardiomyocyte Hypertrophy
Source: Front Pharmacol. 2019 Jan 8;9:1519. doi: 10.3389/fphar.2018.01519 (PMC6331469; doi:10.3389/fphar.2018.01519)
Supplement: Supplementary file 1 [file Table_1.docx]

**Supplementary material**

**SIRT6 suppresses NFATc4 expression and activation in cardiomyocyte hypertrophy**

**Running title:** SIRT6 suppresses NFATc4 in cardiomyocyte hypertrophy

Zhenzhen Li ^a, #^, Xiaoying Zhang ^b, #^, Zhen Guo ^a^, Yao Zhong ^a, c^, Panxia Wang ^a^, Jingyan Li ^a^, Zhuoming Li ^a,^ *, Peiqing Liu ^a,^ *

^a^ Department of Pharmacology and Toxicology, School of Pharmaceutical Sciences; National and Local United Engineering Lab of Druggability and New Drugs Evaluation; Guangdong Provincial Key Laboratory of New Drug Design and Evaluation, Sun Yat-Sen University, Guangzhou, PR China

^b^ Department of Pharmacology, School of Medicine, Xizang Minzu University, Shaanxi, PR China

^c^ Department of Cardiology, Third people's Hospital of Dongguan, Dongguan, PR China

**^#^**Zhenzhen Li and Xiaoying Zhang contributed equally to this work.

*Corresponding authors: Prof. Peiqing Liu & Dr. Zhuoming Li

Address: Department of Pharmacology and Toxicology, School of Pharmaceutical Sciences, Sun Yat-sen University (Higher Education Mega Center), 132**^#^** East Wai-huan Road, Guangzhou 510006, Guangdong, PR China.

Fax: +86 20 39943026

E-mail address: liupq@mail.sysu.edu.cn (P. Liu); lizhm5@mail.sysu.edu.cn (Z. Li)

**Table S1 Primer sequences for qRT-PCR**

| **Gene** | **Sequences** | **Species** |
| --- | --- | --- |
| **SIRT6** | Forward:5'-GCCGTCTGGTCATTGTCA-3'  Reverse:5'-AGCCTTGGGTGCTACTGG-3' | Rattus norvegicus |
| **BNP** | Forward:5'-GGAAGTCAACCCGTCTCA-3'  Reverse:5'-AGCCCTCAGTTTGCTTTT-3' | Rattus norvegicus |
| **NFATc4** | Forward:5'-TCTTAACCTGGGTGCTGGCT-3'  Reverse:5'-CACACCCTCAGTCCCTTTCAA-3' | Rattus norvegicus |
| **β-actin** | Forward:5'-TCGTGCGTGACATTAAAGAG-3'  Reverse:5'-ATTGCCGATAGTGATGACCT-3' | Rattus norvegicus |

**Abbreviations:** SIRT6, Sirtuin 6; BNP, brain natriuretic polypeptide; NFATc4, nuclear factor of activated T cells c4

**Table S2 Small interference RNAs**

| **Name** | **Primer Sequences** |
| --- | --- |
| **SIRT6** | Forward:5'-GCCGUCUGGUCAUUGUCAATT-3'  Reverse:5'-UUGACAAUGACCAGACGGCTT-3' |
| **NFATc4** | Forward:5'-GGAGUCUGAACUUAAUGAATT-3'  Reverse:5'-UUCAUUAAGUUCAGACUCCTT-3' |

**Table S3 Echocardiographic parameters from all groups of rats**

| **Parameters** | **Sham**  **(*n* = 6)** | **AAC**  **(*n* = 6)** |
| --- | --- | --- |
| **LVAW_d_ (mm)** | 1.48 ± 0.05 | 2.52 ± 0.29** |
| **LVAW_s_ (mm)** | 2.12 ± 0.10 | 3.84 ± 0.13*** |
| **LVID_d_ (mm)** | 7.47 ± 0.22 | 5.73 ± 0.56* |
| **LVID_s_ (mm)** | 5.02 ± 0.19 | 2.80 ± 0.19*** |
| **LVPW_d_ (mm)** | 1.73 ± 0.05 | 2.57 ± 0.20* |
| **LVPW_s_ (mm)** | 2.52 ± 0.09 | 3.49 ± 0.22** |
| **EF (%)** | 59.56 ± 1.75 | 80.46 ± 2.28*** |
| **FS (%)** | 32.84 ± 1.24 | 50.12 ± 2.63*** |
| **CO (mL)** | 59.13 ± 4.11 | 48.46 ± 8.22 |
| **SV (mL)** | 176.8 ± 10.72 | 142.1 ± 30.34 |
| **HR (bpm)** | 335.8 ± 17.42 | 379.2 ± 44.8 |

**Abbreviations:** AAC, abdominal aortic constriction; LVAW, left ventricular anterior wall thickness; LVID, left ventricular internal diameter; LVPW, left ventricular posterior wall thickness; -d, diastolic; -s, systolic; EF, ejection fraction; FS, fractional shortening; CO, cardiac output; SV, stroke volume; HR, heart rate. All values are presented as means ± SEM. **P* < 0.05, ***P* < 0.01, ****P* < 0.001 *vs.* normal saline (NS) group.


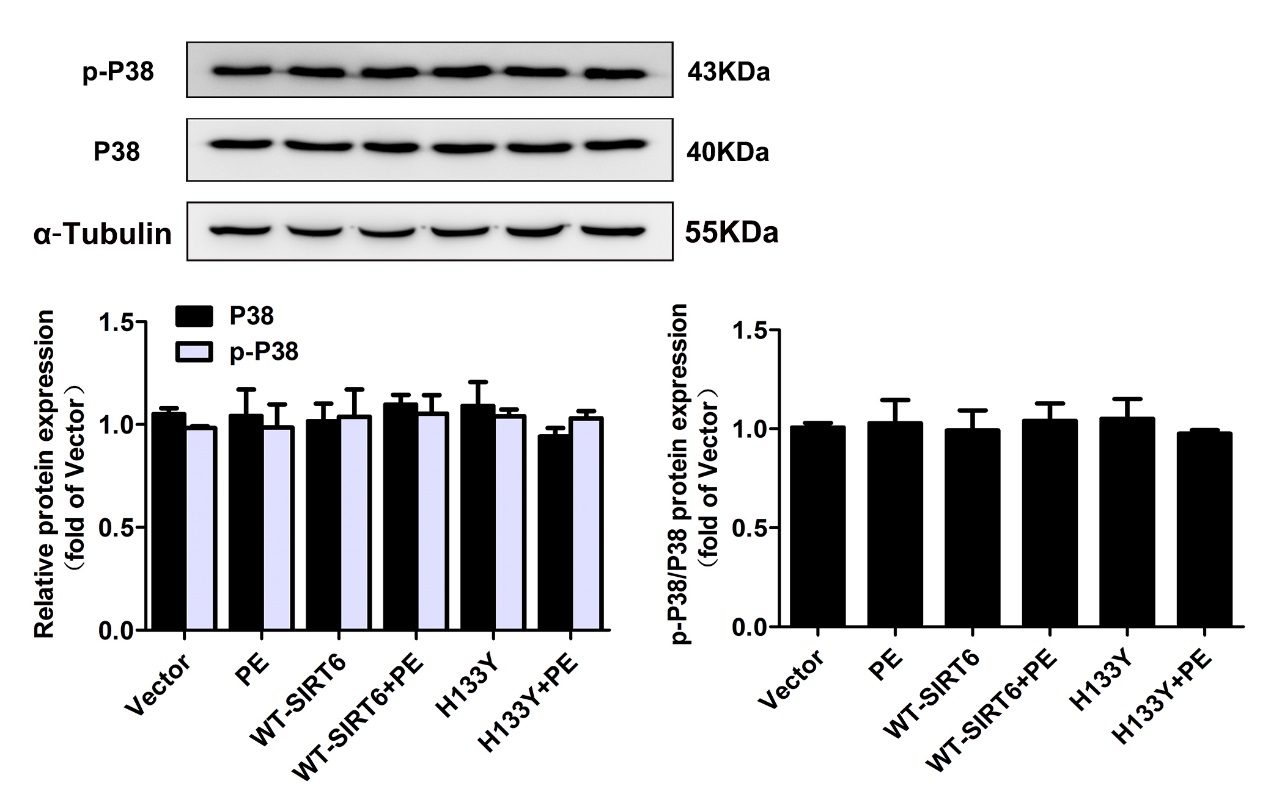


**Fig. S1. Change of p38 expression in SIRT6 overexpression.** NRCMs were transiently transfected with plasmid encoding the wildtype SIRT6 (WT-SIRT6) and mutant of SIRT6 (H133Y) for 48h. Western blot analysis was conducted to determine the protein expression of p38. Data were normalized by α-tubulin for protein expression and presented as mean ± SEM, *n* = 4.

**

**

**Fig. S2. Changes of miR-29a and miR-133a expression in SIRT6 overexpression.** NRCMs were transiently transfected with plasmid encoding the wildtype SIRT6 (WT-SIRT6) for 48h and the expression of miR-29a and miR-133a were determined by qRT-PCR. The data were presented as mean ± SEM. ***P* < 0.01 *vs.* Vector. *n* = 4.

A


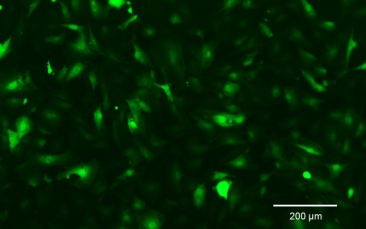


B


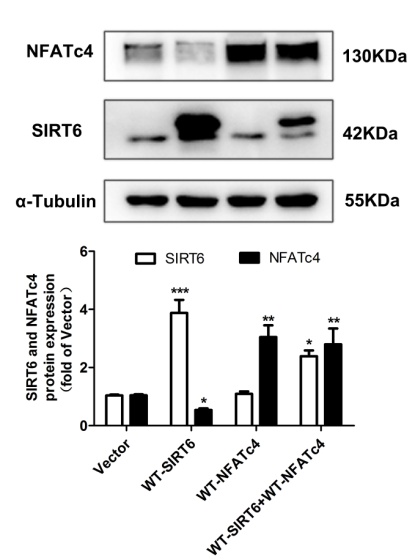


**Fig. S3. The transfection efficiency of cardiomyocytes. (A)** Cells were transfected with GFP-SIRT1 plasmid for 48 h using Lipofectamine 2000. The image was captured by fluorescence microscope. **(B)** Cells were transfected with WT-SIRT6 and WT-NFATc4 plasmids for 48 h using Lipofectamine 2000. The protein expression of NFATc4 and SIRT6 were measured by Western blotting. The data were presented as mean ± SEM. **P* < 0.05, ***P* < 0.01, ****P* < 0.001 *vs.* Vector. *n* = 4.
